# Is working from home changing the meaning of work?

**DOI:** 10.1371/journal.pone.0340452

**Published:** 2026-01-27

**Authors:** Sebastian Bähr, Bernad Batinic, Matthias Collischon

**Affiliations:** 1 Institute for Employment Research, Nürnberg, Germany; 2 Institute of Psychology, JKU Linz, Linz, Austria; 3 IZA, Bonn, Germany; 4 LASER, Nürnberg, Germany; Wroclaw University of Economics and Business: Uniwersytet Ekonomiczny we Wroclawiu, POLAND

## Abstract

Especially since the COVID-19 pandemic, working from home (WFH) has become a common practice in the workplace. This raises the question of whether WFH changes the non-monetary benefits of work, such as job quality or social contacts. Thus, in this article, we investigate how working from home affects Jahoda’s latent functions of employment as well as job quality measures. To this end, we use panel data from the German Panel Study Labour Market and Social Security (PASS) and estimate the effects of changing work patterns on the aforementioned outcomes. Our findings reveal basically no effects of WFH on job quality measures and latent benefits. This, in contrast to anecdotal evidence, implies that WFH does not harm psychological well-being.

## 1 Introduction

Working from home (WFH) has become one of the most significant changes to the world of work in recent decades. What was initially intended as a temporary response to the coronavirus disease 2019 (COVID-19) quickly turned into a permanent practice and a new reality [1,2]. In 2019, only about 7% of U.S. employees regularly worked from home. By 2023, however, nearly one in four paid workdays were performed remotely, and 15% of employees were working entirely remotely. According to a 2020 survey, 66% of workers stated they would like to WFH at least one day per week, and almost 45% would prefer three or more days [3].

For many employees, the flexibility of WFH has made a lasting difference in both their professional and personal lives. While some came to appreciate benefits such as increased flexibility and time savings, others experienced WFH as a burden [4,5]. These mixed experiences are also reflected in empirical research, which highlights both opportunities and challenges of remote work. On the one hand, WFH can provide greater autonomy, reduced commuting time and costs, and higher job satisfaction and motivation [6–9]. On the other hand, it is often associated with social isolation [10,11], difficulties in separating work and private life, increased work hours [4], and an inadequate working environment [12].

From an organizational perspective, WFH is also associated with ambivalent consequences. Many companies are adapting to this trend, increasingly advertising remote work options and incorporating flexible work concepts into office planning [13]. At the same time, employers often voice concerns that WFH may reduce productivity (but see also [3, 14–16]), weaken employee loyalty, and diminish opportunities for collaboration, creativity, and innovation [17,18]. As a result, negotiations are underway between employees and employers regarding the optimal mix of in-office and remote work. Some employers even want to terminate agreements on WFH altogether and call on their employees to return to in-person work. A particularly prominent example of this is President Trump’s presidential memorandum, Return to In-Person Work [19]. As a result, numerous ministries are inclined to reduce remote work.

Beyond the individual and organizational levels, WFH also has a broader social impact. Reduced commuting can lower fossil fuel consumption, air pollution, and CO₂ emissions, while geographic flexibility may relieve traffic in metropolitan areas and increase quality of life [20–23]. Although these externalities are essential, the more fundamental psychological question is how WFH affects the meaning of work.

Unlike previous debates about productivity or environmental benefits, the present study focuses on how WFH changes employees’ access to the psychological and social functions that work provides. We are the first to apply Jahoda’s concept of work’s latent functions [24,25], an established socio-psychological framework, in the context of WFH. We thus contribute to the literature on the interplay between workplace factors and employee well-being by investigating how within-person changes in WFH affect various socio-psychological measures. To this end, we use large-scale German Panel data and apply fixed-effects regression models that account for time-invariant unobserved heterogeneity.

## 2 Theoretical background and literature review

### 2.1 Jahoda’s latent deprivation model

Work has always been more than earning a living. As Jahoda’s latent deprivation model [24,25] highlights, employment fulfills five latent functions beyond income: providing time structure, social contacts, collective purpose, status, and activity. Her model, rooted in the seminal Marienthal study [26], shows that when people lose their jobs, they not only suffer financial strain but also deprivation of these latent functions, leading to declines in well-being and health. Importantly, subsequent research has shown that even among the employed, occupations and work contexts differ in the extent to which they provide access to these latent functions [27]. Therefore, changes in the conditions under which work is carried out may alter people’s access to the latent benefits of employment and, in turn, their well-being.

### 2.2 Working from home: A brief literature review

Beyond this theoretical perspective, a rapidly growing body of empirical research has examined how working from home relates to employees’ job quality and well-being. Several studies report positive associations between WFH and job satisfaction, work–life balance, and general well-being, often attributing these benefits to increased flexibility, reduced commuting time, and higher perceived autonomy. Other studies, however, emphasise potential risks of WFH, including social isolation, blurred boundaries between work and private life, longer working hours, and increased strain [10–12]. Evidence from a meta-analysis [28] and a subsequent integrative review [29] suggests that mixed findings reflect small average effects and systematic mediation and moderation. Autonomy appears to be a key mediator, and effects vary with boundary conditions such as the intensity and organisation of remote work. Taken together, this literature suggests that WFH can both improve and impair essential aspects of job quality and psychological health, and that the overall impact likely depends on how remote work is organised and experienced. But most existing studies focus on global indicators of well-being or work attitudes. They rely on cross-sectional or self-selected samples and rarely investigate the socio-psychological mechanisms through which WFH might affect the meaning of work. In particular, it remains unclear whether changes in WFH arrangements alter employees’ access to the latent functions of employment in Jahoda’s sense and how such changes relate to mental health, social integration, and overall well-being.

### 2.3 Latent functions and contemporary theories of work and well-being

Jahoda’s latent functions can also be understood in the context of contemporary psychological theories. Self-determination theory (SDT) [30] emphasizes the universal needs for autonomy, competence, and relatedness, which closely align with Jahoda’s functions of time structure, social contact, and collective purpose. Similarly, the conservation of resources theory (COR) [31] conceptualizes stress and well-being as outcomes of the loss or gain of valuable resources, resonating with Jahoda’s view that employment provides latent resources beyond financial income. From an organizational psychology perspective, the Job Demands–Resources model (JD-R) [32] offers a dynamic framework for understanding how the availability or absence of job resources affects motivation and health. Seen in this light, Jahoda’s model can be regarded as an early articulation of psychological need fulfillment at work, which remains highly relevant in contemporary debates about remote work and changing work environments.

Against this background, the spread of WFH may disrupt the balance of latent functions provided by employment. While employees continue to receive income, they may experience reduced access to social connections, collective purpose, or daily structure, which could ultimately lead to alienation from work. In the long run, this may also have adverse effects on health and well-being [27].

Building on this framework, the present study investigates whether WFH changes the meaning of work by altering employees’ access to its latent functions and how this, in turn, affects their mental health, social integration, and well-being.

### 2.4 Research questions

Based on prior literature and theoretical considerations, we expect working from home to affect the latent functions of employment and psychological well-being, as it induces far-reaching changes in how work is conducted; however, the direction of the effect remains unclear. On the one hand, WFH could imply greater autonomy at work and allow for more structure by reducing time spent commuting. On the other hand, WFH might lead to a loss of structure, as the private and work spheres are no longer strictly distinguished, and to a loss of social connections, with adverse effects on well-being. We will test which effect dominates in the empirical estimations. This leads to the following two research questions:

RQ1: Does starting or stopping WFH change employees’ access to the latent functions of employment?
RQ2: Does starting or stopping WFH affect job satisfaction, psychological health, and social integration?

## 3 Method

### 3.1 Data

We use data from waves 14–16 (2020–2022) of the household panel study “Labor Market and Social Security” (PASS) in our analysis. PASS is a survey conducted by the IAB (Institute for Employment Research of the Federal Employment Agency, Germany) and designed to facilitate research on the labor market and poverty in Germany [33]. PASS includes rich information on individuals’ employment situations, socioeconomic characteristics, and household contexts, enabling detailed subgroup analyses of specific employment groups. It consists of two large random samples: one from the German population and one from welfare benefit recipients.

Ethical review and approval were not required for the study involving human participants, in accordance with local legislation and institutional requirements. The patients/participants provided their written informed consent to participate in this study.

### 3.2 Analysis sample

From an initial sample of 18,132 participants from 2020 to 2022, we focus on the 5,786 office workers and retain 3,734 participants in the analysis sample. This loss of 2,052 individuals is due to conditioning on non-missing LaMB factor information, well-being outcomes, and control variables. Please note that the case numbers in the analyses could vary due to missing values in specific outcomes.

### 3.3 Measures

#### 3.3.1 *The LaMB module.*

Latent and Manifest Benefits of Work were measured with the German version of the shortened Latent and Manifest Benefits of Work Scale (LaMB) from [34]. Based on two larger empirical studies (*N* = 1054; *N* = 677), the scale’s authors conclude that their version of LaMB is an economical (i.e., cost-effective) instrument with satisfactory psychometric properties. The scale consists of 18 items, where the five latent benefits (collective purpose, social contact, status, activity, and time structure) and financial strain, a (negatively phrased) manifest benefit, are each measured with three items. Respondents scored each item on a seven-point Likert scale (“completely disagree” to “completely agree”; see Table A1 of the appendix for the exact wording).

We use the scale to construct standardized summary indices for the five latent factors.

#### 3.3.2 *WFH.*

Since the beginning of the COVID-19 lockdowns in Germany, PASS has asked respondents whether they work primarily from home. We use this item to construct a WFH variable. We further generated variables indicating whether individuals transitioned to WFH (i.e., reported working out of home in one survey wave and then switched to remote work in the next wave, or vice versa).

#### 3.3.3 *Well-being measures.*

We use three variables to measure well-being: job satisfaction, psychological health, and social integration. *Job satisfaction* is self-assessed using the question “How satisfied are you with your work currently?” Individuals should then sort themselves on a scale from 0 (not at all satisfied) to 10 (very satisfied) [35]. *Psychological health* is measured by asking, “How strongly have you been affected by mental problems, such as fear, dejection, or irritability, in the past four weeks?” Response categories range from 1 (not at all) to 5 (extremely). We reversed the scale for the analysis; thus, a lower score means lower mental health. The scale originates from the SF-12 short survey on health-related quality of life [35]. *Social Integration* is the self-assessment in response to the question, “One may feel integrated into everyday social life and be a real part of society, or one may feel excluded. What about you?” Individuals can sort themselves from 1 (excluded from social life) to 10 (integrated into social life). The scale captures social exclusion and participation [35], two multifaceted concepts that are often discussed in the context of WFH.

#### 3.3.4 *Control variables.*

The PASS also includes a wide range of potential control variables. In our estimations, we control for age, years of education, monthly gross individual labor income, household composition, partnership status, job tenure, occupation (as 2-digit-indicator variables), industry (as 2-digit-indicator variables), whether individuals are on a short-term work allowance scheme, job changes during the last survey wave, interview mode, and COVID-wave indicators. Table 1 provides an overview of all variables used in the analysis.

**Table 1:** Sample descriptives.

		Median	Mean	Std. dev.	Min	Max
Dependent variables	Collective Purpose	0	0.25	0.73	−1.93	1.65
	Social Contact	0	0.14	0.70	−1.53	1.73
	Status	0	0.13	0.68	−3.11	1.19
	Activity	1	0.41	0.56	−2.34	0.86
	Time Structure	0	0.04	0.65	−2.48	1.19
	Financial Strain	−1	−0.37	0.76	−1.49	1.47
	Job satisfaction (0–10)	8	7.30	1.80	0.00	10.00
	Subjective mental health (1–5)	4	3.67	1.17	1.00	5.00
	Social Integration (1–10)	8	7.36	1.91	1.00	10.00
Independent Variables	Age (years)	42	43.00	11.84	18.00	65.00
	Female (0/1)	1	0.55	0.50	0.00	1.00
	Years of education	13	13.57	2.89	7.00	21.00
	Individual gross income (EUR)	2,500	2,936.09	3,033.02	0.00	100,000
	Partner in Household (0/1)	1	0.57	0.50	0.00	1.00
	Child under 15 in household (0/1)	0	0.30	0.46	0.00	1.00
	Job tenure (months)	37	75.67	97.00	0.00	571
	Job change (0/1)	0	0.09	0.29	0.00	1.00
	Short-time-allowance (0/1)	0	0.06	0.24	0.00	1.00
	Change in urbanity level (0/1)	0	0.04	0.20	0.00	1.00
	Face-to-face interview mode (vs. telephone mode)	0	0.02	0.15	0.00	1.00
	Survey quarter	2	1.66	0.72	1.00	3.00
	Survey year	2021	2021.19	0.76	2020	2022

Source: PASS W14-W16.

### 3.4 Methods

To estimate the effects of interest, we estimate the following estimation equation via ordinary least squares (OLS) regressions:


$$Y_{it}=\beta_{\text{0}}+\beta_{\text{1}}wf{h^{\prime }}_{it}+\gamma {X^{\prime }}_{it}+\in_{it}$$

**Fig 1:**
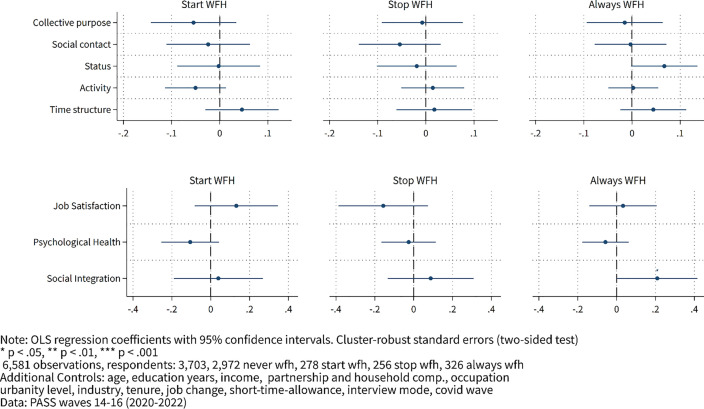
Effects of WFH on latent factors and well-being measures.

Where Y is the outcome of interest (either the latent functions of employment or well-being measures), wfh is a set of indicator variables for WFH (start WFH, stop WFH, always WFH with the reference category being never WFH), and X is a set of control variables (as described in the data section). As we use panel data, we have information on individuals *i* in time periods *t*, as denoted by the subscripts. Fig 1 plots the estimates of $\beta_{\text{1}}$, i.e., the partial correlation between working-from-home measures and *t*he outcomes of interest.

**Fig 2:**
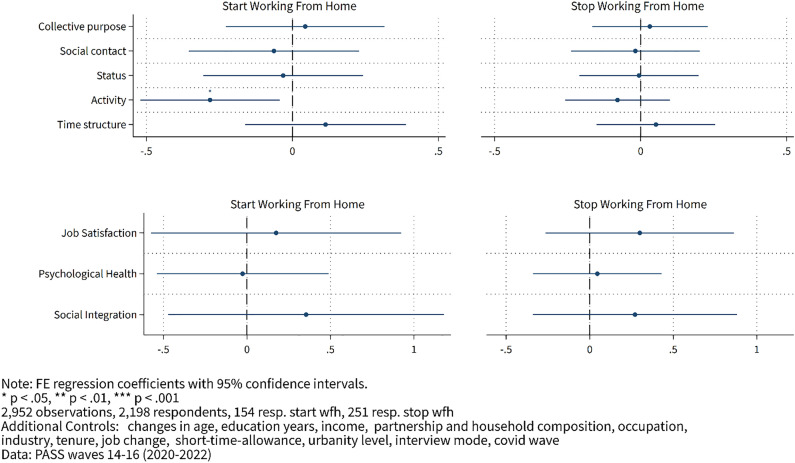
Effects of WFH on latent factors and well-being measures – fixed effects results.


In Fig 2, we estimate individual fixed effects regressions in the following form:

$$Y_{it}=\alpha_{i}+\lambda wf{h^{\prime }}_{it}+\delta {X^{\prime }}_{it}+\varepsilon_{it}$$

where $\alpha_{i}$ are individual fixed effects to account for time-constant heterogeneity between individuals. Introducing fixed effects allows us to use only time-varying information in the estimations; thus, we estimate no effect for always WFH.

## 4 Results

### 4.1 Main results

In our principal analysis using data from the Panel Labour Market and Social Security (PASS) [33], we investigate the effects of WFH on Jahoda’s five latent functions, job satisfaction, psychological health, and social integration (see the Data Section at the end of this article for more information on the data).

We begin by estimating the correlations between latent functions of employment and WFH in the upper panel of Fig 1. We estimate partial effects of three types of arrangements using OLS regression: i) starting WFH, ii) stopping WFH, or iii) always WFH. The omitted reference category is never WFH; thus, all comparisons are marginal effects relative to individuals who never WFH. As can be seen, WFH does not correlate significantly with either latent function, except for status for those who always WFH – but this could be either by chance (due to the large number of tests we conduct) or because those who always WFH hold higher positions, even within occupation-categories, that are not accounted for by occupation and income controls. Overall, we conclude that WFH neither increases nor impairs the fulfillment of the latent benefits of work.

Turning to the well-being outcomes in the lower panel of Fig 1, we observe evidence consistent with the effects on the latent functions: WFH neither significantly improves nor worsens job satisfaction or psychological health. For social integration, we find no correlation with starting or stopping WFH; however, contrary to the literature, we observe a significant positive association between WFH and social integration among those who always WFH. Again, this could be due to unaccounted heterogeneity, e.g., well-integrated workers choosing to WFH.

Overall, the results from these estimates indicate no noteworthy association between WFH and either latent employment functions or measures of well-being. These findings are even more striking as the estimations could be biased by unobserved heterogeneity. However, if this were the case, our prior would be to find significant effects even in the absence of any, as we would not expect a bias toward zero.

In addition to the OLS regression results shown in Fig 1 and previously discussed, we also conducted individual-level fixed-effects estimations. These estimates entail a loss of statistical power because we do not account for individual-level variation in this regression. Still, they have the advantage of accounting for time-constant unobserved heterogeneity. In this case, we cannot estimate the effects of the “always WFH” category, as fixed-effects estimation requires variation over time within individuals, which is absent for individuals who always WFH.

Fig 2 shows the results of the fixed effects estimations. As shown, the results largely mirror those in Fig 1. However, because the estimates are relatively imprecise (as indicated by the large confidence bands) and because we conduct many tests, our baseline conclusion from these estimates is that latent functions appear relatively invariant to WFH.

In the lower panel of Fig 2, we again investigate the impact of WFH on well-being. Again, we observe no statistically significant effects. Job satisfaction, psychological health, and social integration do not seem to depend on whether employees WFH or return to the office. These results are consistent with other results from Germany [36], Italy [37] that also report null effects.

As a robustness check, we recalculate our estimation using propensity score matching. We match on all variables that we use as control variables in our baseline estimations to estimate the probability of i) starting WFH, ii) stopping WFH, or iii) always WFH in three separate logit regressions, imposing common support and using a caliper of 0.01 with radius matching. We then use the weights obtained from these estimations to repeat our OLS analysis. Appendix Figure A1 shows the results. As shown, the results are broadly consistent with our main findings, thereby demonstrating the robustness of our findings.

### 4.2 Heterogeneity analysis

Our main findings indicate minimal effects of WFH on latent employment functions and well-being outcomes. However, the average effects for the whole sample may conceal effects for specific groups that may be heterogeneously affected by WFH.

We begin by investigating gender differences. WFH may blur the boundaries between work and household duties, potentially adversely affecting men and women. Appendix Figures A1 and A2 present the results for men and women, respectively, using OLS and FE regressions. In neither case do we find significant effects of WFH, indicating no pronounced gender difference in the impact.

Next, we investigate differences between individuals with and without children in the household in Appendix Figures A3 and A4, again displaying the OLS and FE results. It could be the case that WFH affects the outcome measures adversely, as, on the one hand, caring for children in addition to working from home might induce conflicts and stress, but, on the other hand, it could also make it easier to reconcile childcare and work duties if one does not need to commute to work. However, the results show no difference between parents and non-parents (this finding also holds when further differentiating by gender; not shown for brevity).

Lastly, WFH could adversely affect outcomes of interest regarding job characteristics. Here, we investigate differences between individuals with and without managerial duties, as managing employees may be more difficult and thus more stressful in a WFH setting than in the office. Appendix Figures A5 and A6 show the results. Again, we observe no significant effects. Overall, we find little evidence that WFH affects latent benefits or well-being measures. These subgroup analyses provide further evidence that we do not miss specific effects by examining only full-sample averages. Appendix Table A2 further presents the number of cases used in our subgroup analysis. Because the number of cases is always relatively large, we can confidently conclude that a small number of cases does not drive our results; instead, there are likely no, or at least no significant, effects of WFH on the outcomes we investigate.

## 5 Discussion

We contribute to the current debate about the merits and perils of WFH by applying Jahoda’s concept of the latent functions of work to changes in WFH arrangements. Addressing RQ 1, we find no evidence that starting or stopping WFH systematically alters employees’ access to these vital psychological functions. Addressing RQ 2, we likewise find no meaningful differences in job satisfaction, psychological health, or social integration across work settings. Overall, our results suggest that WFH fulfills the latent functions of employment no differently from work at the firm, and that employees’ well-being is primarily affected by work location.

These results may surprise, given that WFH is often claimed to be an attractive job characteristic for which employees are willing to even forgo higher wages [38,39]. However, the COVID-19 pandemic introduced large parts of the workforce to – often mandatory – WFH. Thus, the usual sorting of employees into jobs that allow or disallow WFH, which occurred before and after the pandemic, is absent from our data. If people can choose WFH deliberately, we would expect the effects to be more advantageous for individuals, but never more negative. The pandemic also furthered the widespread adoption of technical tools that facilitated video communication [40] and normalized their use in day-to-day work, resulting in greater WFH acceptance than before the pandemic.

Taken together, our findings indicate that WFH is neither inherently beneficial nor harmful to employees’ well-being, but it increases the options available to employees and firms. Jobs performed from home can be as meaningful as jobs performed at the office, and meaningful work remains a key factor of job performance [41,42]. In line with previous findings on the relationship between WFH and job characteristics [7]. This result further indicates that, from employees’ perspective, the location of work has no impact on their psychological well-being. Our results, therefore, speak to the managerial debate on return-to-office mandates and the optimal extent of WFH [37,43]: instead of assuming that systematic gains or losses in well-being arise solely from work location, attention should focus on how remote work is designed, supported, and integrated into everyday work practices.

## 6 Limitations

Using nationally representative large-scale German data, we can provide insights into how the increase in WFH in the wake of the COVID-19 pandemic influenced employees’ psychological well-being.

Given the large number of observations from a probability sample, individual heterogeneity is unlikely to bias our results systematically. On the contrary, our results are robust across cross-sectional and longitudinal designs and across many subgroups, with no differences in WFH by gender, parental status, or managerial status, while controlling for income, job tenure, industry, and occupation.

We can provide evidence only for German office workers, using our unique data that combine detailed labor market biographies, work-from-home status, and psychological well-being. The distinctive cultural, economic, and regulatory characteristics of the national context make generalizing from any study relying on data from a single country problematic. However, regarding the WFH propensity during the COVID-19 pandemic, Germany was reasonably similar to other Western countries [44–46]. Additionally, the latent deprivation model, as a sociopsychological concept, has been demonstrated to be effective in various international contexts [47], which gives us confidence that our results are generalizable.

While we can address substantial heterogeneity stemming from sociodemographic background and employment histories, our data lack details on the extent of WFH. While using WFH as a binary indicator is established in the literature [48], we lack more detailed data on WFH. We do not know, for example, whether WFH occurred once a week or daily, or how many months the employee was WFH between the annual interviews. This unobserved heterogeneity might thus influence our results. Future research should adopt a finer-grained view of working from home and, if possible, investigate how the intensity of WFH or fluctuations in WFH duration over time affect well-being outcomes.

While controlling for time-constant confounding, time-varying confounders may still impact our FE analysis. Although we control for a range of sociodemographic characteristics and job changes, we lack information on more subtle work-related factors, such as workplace policies or the career prospects of remote workers relative to those of on-site workers. These factors might influence both an office worker’s decision on where to work and their overall well-being.

Given that we can only compare 2020−2022, we cannot make claims about periods before the COVID-19 pandemic, when WFH was less common, or after, when it is much more commonplace. WFH in both periods is driven primarily by employees’ choice, with many employers seeking to return their workforce to the office. Thus, the analysis of these periods would be compromised by sorting and selection into WFH. By exploiting the natural social experiment of the COVID-19 pandemic and a longitudinal research design, our analysis suffers considerably less from selection bias. It provides novel insights into the influence WFH has on employees’ well-being.

## Supporting information

S1 Appendix Online Appendix. (PDF)

## References

[1] Tunk N, Kumar AA. Work from home - A new virtual reality. Curr Psychol. 2022;1–13. doi: 10.1007/s12144-021-02660-0 35125850 PMC8799454

[2] Jain T, Currie G, Aston L. COVID and working from home: Long-term impacts and psycho-social determinants. Transp Res Part A Policy Pract. 2022;156:52–68. doi: 10.1016/j.tra.2021.12.007

[3] Barrero JM, Bloom N, Davis SJ. Why Working From Home Will Stick. SSRN J. 2020. doi: 10.2139/ssrn.3741644

[4] Wang B, Liu Y, Qian J, Parker SK. Achieving Effective Remote Working During the COVID-19 Pandemic: A Work Design Perspective. Appl Psychol. 2021;70(1):16–59. doi: 10.1111/apps.12290 33230359 PMC7675760

[5] Peters SE, Dennerlein JT, Wagner GR, Sorensen G. Work and worker health in the post-pandemic world: a public health perspective. Lancet Public Health. 2022;7(2):e188–94. doi: 10.1016/S2468-2667(21)00259-0 35122760 PMC8809900

[6] Becker C, Soucek R, Göritz AS. Activity-based working: How the use of available workplace options increases perceived autonomy in the workplace. Work. 2022;73(4):1325–36. doi: 10.3233/WOR-210767 36093664

[7] Schulze J, Krumm S, Eid M, Müller H, Göritz AS. The relationship between telework and job characteristics: A latent change score analysis during the COVID-19 pandemic. Appl Psychol. 2023;73(1):3–33. doi: 10.1111/apps.12461

[8] Niebuhr F, Borle P, Börner-Zobel F, Voelter-Mahlknecht S. Healthy and Happy Working from Home? Effects of Working from Home on Employee Health and Job Satisfaction. Int J Environ Res Public Health. 2022;19(3):1122. doi: 10.3390/ijerph19031122 35162145 PMC8834350

[9] Prodanova J, Kocarev L. Employees’ dedication to working from home in times of COVID-19 crisis. MD. 2021;60(3):509–30. doi: 10.1108/md-09-2020-1256

[10] Tull MT, Edmonds KA, Scamaldo KM, Richmond JR, Rose JP, Gratz KL. Psychological Outcomes Associated with Stay-at-Home Orders and the Perceived Impact of COVID-19 on Daily Life. Psychiatry Res. 2020;289:113098. doi: 10.1016/j.psychres.2020.113098 32434092 PMC7252159

[11] Philpot LM, Ramar P, Roellinger DL, Barry BA, Sharma P, Ebbert JO. Changes in social relationships during an initial “stay-at-home” phase of the COVID-19 pandemic: A longitudinal survey study in the U.S. Soc Sci Med. 2021;274:113779. doi: 10.1016/j.socscimed.2021.113779 33639395 PMC7895700

[12] Davis KG, Kotowski SE, Daniel D, Gerding T, Naylor J, Syck M. The Home Office: Ergonomic Lessons From the “New Normal”. Ergon Des. 2020;28(4):4–10. doi: 10.1177/1064804620937907 40963944 PMC12439588

[13] Myerson J, Ross P. Unworking. The reinvention of the modern office. London: Reaktion; 2022.

[14] Bloom N, Liang J, Roberts J, Ying ZJ. Does Working from Home Work? Evidence from a Chinese Experiment *. Q J Econ. 2014;130(1):165–218. doi: 10.1093/qje/qju032

[15] Bloom N, Han R, Liang J. Hybrid working from home improves retention without damaging performance. Nature. 2024;630(8018):920–5. doi: 10.1038/s41586-024-07500-2 38867040 PMC11208135

[16] Bloom N, Bunn P, Mizen P, Smietanka P, Thwaites G. The Impact of Covid-19 on Productivity. Rev Econ Stat. 2025;107(1):28–41. doi: 10.1162/rest_a_01298

[17] Maksim M, Śliwicki D. Factors Determining Employee Loyalty During the COVID-19 Pandemic. Sustainability. 2025;17(1):303. doi: 10.3390/su17010303

[18] Tønnessen Ø, Flåten B-T. Work from home and collective creativity: Exploring the experiences of IT professionals. Cog Bus Manag. 2023;10(3). doi: 10.1080/23311975.2023.2262219

[19] The White House. Return to In-Person Work. 2025 [updated 21 Jan 2025; cited 25 Feb 2025]. Available from: https://www.whitehouse.gov/presidential-actions/2025/01/return-to-in-person-work/

[20] Navaratnam S, Jayalath A, Aye L. Effects of Working from Home on Greenhouse Gas Emissions and the Associated Energy Costs in Six Australian Cities. Buildings. 2022;12(4):463. doi: 10.3390/buildings12040463

[21] McPhail R, Chan XW, May R, Wilkinson A. Post-COVID remote working and its impact on people, productivity, and the planet: an exploratory scoping review. Int J Hum Resour Manag. 2023;35(1):154–82. doi: 10.1080/09585192.2023.2221385

[22] Choudhury P, Foroughi C, Larson B. Work-from-anywhere : The productivity effects of geographic flexibility. Strat Manag J. 2020;42(4):655–83. doi: 10.1002/smj.3251

[23] Harantová V, Hájnik A, Kalašová A, Figlus T. The Effect of the COVID-19 Pandemic on Traffic Flow Characteristics, Emissions Production and Fuel Consumption at a Selected Intersection in Slovakia. Energies. 2022;15(6):2020. doi: 10.3390/en15062020

[24] Jahoda M. Work, employment, and unemployment: values, theories, and approaches in social research. Am Psych. 1981;36:184–91.

[25] Jahoda M. Employment and unemployment. A social-psychological analysis. Cambridge University Press; 1982.

[26] Jahoda M, Lazarsfeld PF, Zeisel H, Fleck C. Marienthal. The sociology of an unemployed community. New Brunswick (NJ): Transaction; 1933.

[27] Batinic B, Selenko E, Stiglbauer B, Paul KI. Are workers in high-status jobs healthier than others? Assessing Jahoda’s latent benefits of employment in two working populations. Work Stress. 2010;24(1):73–87. doi: 10.1080/02678371003703859

[28] Gajendran RS, Harrison DA. The good, the bad, and the unknown about telecommuting: meta-analysis of psychological mediators and individual consequences. J Appl Psychol. 2007;92(6):1524–41. doi: 10.1037/0021-9010.92.6.1524 18020794

[29] Allen TD, Golden TD, Shockley KM. How Effective Is Telecommuting? Assessing the Status of Our Scientific Findings. Psychol Sci Public Interest. 2015;16(2):40–68. doi: 10.1177/1529100615593273 26403188

[30] Ryan RM, Deci EL. Self-determination theory and the facilitation of intrinsic motivation, social development, and well-being. Am Psychol. 2000;55(1):68–78. doi: 10.1037//0003-066x.55.1.68 11392867

[31] Hobfoll SE. Conservation of resources. A new attempt at conceptualizing stress. Am Psychol. 1989;44(3):513–24. doi: 10.1037//0003-066x.44.3.513 2648906

[32] Bakker AB, Demerouti E. The Job Demands-Resources model: state of the art. J Manag Psychol. 2007;22(3):309–28. doi: 10.1108/02683940710733115

[33] Trappmann M, Bähr S, Beste J, Eberl A, Frodermann C, Gundert S. Data Resource Profile: Panel Study Labour Market and Social Security (PASS). Int J Epidemiol. 2019;48:1411. 30982900 10.1093/ije/dyz041 PMC6857753

[34] Kovacs C, Batinic B, Stiglbauer B, Gnambs T. Development of a Shortened Version of the Latent and Manifest Benefits of Work (LAMB) Scale. Eur J Psychol Assess. 2019;35(5):685–97. doi: 10.1027/1015-5759/a000434

[35] Müller B, Bähr S, Gundert S, Teichler N, Unger S, Wenzig C. PASS Scales and Instruments Manual. 2020.

[36] Rudolph CW, Zacher H. Working from home: When is it too much of a good thing? Human Resource Dev Quarterly. 2024;36(1):9–47. doi: 10.1002/hrdq.21530

[37] Costi C, Clark A, D’Ambrosio C, Lepinteur A, Menta G. Return-to-office mandates, health and well-being: evidence from a natural experiment. Esch-sur-Alzette. 2024.

[38] Moens E, Verhofstadt E, Van Ootegem L, Baert S. Disentangling the attractiveness of telework to employees: A factorial survey experiment. Int Lab Rev. 2024;163(2):325–48. doi: 10.1111/ilr.12424

[39] He H, Neumark D, Weng Q. Do Workers Value Flexible Jobs? A Field Experiment. J Lab Econ. 2021;39(3):709–38. doi: 10.1086/711226

[40] Vargo D, Zhu L, Benwell B, Yan Z. Digital technology use during COVID-19 pandemic: A rapid review. Human Behav Emerg Tech. 2020;3(1):13–24. doi: 10.1002/hbe2.242

[41] Allan BA, Duffy RD, Collisson B. Task Significance and Performance. J Career Assess. 2016;26(1):172–82. doi: 10.1177/1069072716680047

[42] Han S-H, Sung M, Suh B. Linking meaningfulness to work outcomes through job characteristics and work engagement. Hum Resour Dev Int. 2020;24(1):3–22. doi: 10.1080/13678868.2020.1744999

[43] Flynn S, Ghent AC, Nair V. Determinants and Consequences of Return to Office Policies. 19 Nov 2025.

[44] Aksoy CG, Barrero JM, Bloom N, Davis SJ, Dolls M, Zarate P. Working from Home Around the World. NBER Work Pap Ser. 2022;(30446). doi: 10.3386/w30446

[45] Schade HM, Digutsch J, Kleinsorge T, Fan Y. Having to Work from Home: Basic Needs, Well-Being, and Motivation. Int J Environ Res Public Health. 2021;18(10):5149. doi: 10.3390/ijerph18105149 34067949 PMC8152264

[46] Schifano S, Clark AE, Greiff S, Vögele C, D’Ambrosio C. Well-being and working from home during COVID-19. ITP. 2021;36(5):1851–69. doi: 10.1108/itp-01-2021-0033

[47] Beck V, Warren T, Lyonette C. Is Any Job Better Than No Job? Utilising Jahoda’s Latent Deprivation Theory to Reconceptualise Underemployment. Work Emp Soc. 2024;39(2):404–25. doi: 10.1177/09500170241254794

[48] Senik C, Clark AE, D’Ambrosio C, Lepinteur A, Schröder C. Teleworking and life satisfaction during COVID-19: the importance of family structure. J Popul Econ. 2024;37(1). doi: 10.1007/s00148-024-00979-z

