# Supplementary material for: Is working from home changing the meaning of work?
Source: PLoS One. 2026 Jan 27;21(1):e0340452. doi: 10.1371/journal.pone.0340452 (PMC12843579; doi:10.1371/journal.pone.0340452)
Supplement: S1 Appendix — (PDF) [file pone.0340452.s001.pdf]

# Appendix to "Is working from home changing the meaning of work?"

**TABLE A1** LAMB module

| Variable | Label | LAMB factor (polarity) | | |
|---|---|---|---|---|
| PLA0100a | I often feel that I make a meaningful contribution to society. | 1 | Collective purpose | + |
| PLA0100b | I often feel a valuable part of society. | 1 | Collective purpose | + |
| PLA0100c | I hold a valuable position in society. | 1 | Collective purpose | + |
| PLA0100d | I often meet new people. | 2 | Social contact | + |
| PLA0100e | I often go out and meet with others. | 2 | Social contact | + |
| PLA0100f | I usually have a lot of opportunities to mix with people. | 2 | Social contact | + |
| PLA0100g | My friends usually value my company. | 3 | Status | + |
| PLA0100h | I am often valued by the people around me. | 3 | Status | + |
| PLA0100i | I am usually important to my friends. | 3 | Status | + |
| PLA0100j | I often have nothing to do. | 4 | Activity | - |
| PLA0100k | I often wish I had more things to do to fill up the time in my days. | 4 | Activity | - |
| PLA0100l | There is usually too much spare time in my day. | 4 | Activity | - |
| PLA0100m | My days are usually well organized. | 5 | Time structure | + |
| PLA0100n | I find it useful to structure my time. | 5 | Time structure | + |
| PLA0100o | I have a good balance in my day between responsibilities and free time. | 5 | Time structure | + |
| PLA0100p | I often have enough money to buy treats for myself. | 6 | Financial strain | - |
| PLA0100q | My income usually allows me to do the things I want. | 6 | Financial strain | - |
| PLA0100r | My level of income usually allows me to make plans for the future. | 6 | Financial strain | - |

Source: PASS W14-W16

Note: Items were presented in randomized order.

**TABLE A2** Subgroup Observations

| | Observations | Respondents |
|---|---|---|
| **Total** | 8.459 | 4.217 |
| **Age** | | |
| under 30 | 1.520 | 950 |
| 31-40 | 2.512 | 1.369 |
| 51-60 | 1.686 | 914 |
| 61-70 | 2.164 | 1.089 |
| 71 and older | 577 | 336 |
| **Gender** | | |
| Male | 3.812 | 1.914 |
| Female | 4.647 | 2.303 |
| **Parenthood (Children under the age of 15 present in the household)** | | |
| No Parent | 5.915 | 3.041 |
| Parent | 2.544 | 1.330 |
| **Gender and Parenthood** | | |
| Male - No Parent | 2.711 | 1.430 |
| Male - Parent | 1.101 | 562 |
| Female - No Parent | 3.204 | 1.611 |
| Female - Parent | 1.443 | 768 |
| **Managerial Position** | | |
| No Managerial Position | 5.436 | 2.831 |
| Managerial Position | 3.019 | 1.521 |
| Missing | 4 | 2 |
| **Classification of Occupations** | | |
| Military | 1 | 1 |
| Agriculture, forestry, animal production, and horticulture | 111 | 63 |
| Raw materials extraction, production, and manufacturing | 1.028 | 529 |
| Construction, architecture, surveying, and building technology | 341 | 179 |
| Natural sciences, geography, and computer science | 550 | 278 |
| Transport, logistics, protection, and security | 845 | 477 |
| Commercial services, commodity trade, distribution, hotels, and tourism | 1.153 | 626 |
| Business organisation, bookkeeping, law, and administration | 1.665 | 842 |
| Healthcare and social occupations, teaching, and education | 2.373 | 1.170 |
| Linguistics, literature, humanities, social sciences and economics, media, art, culture, and design | 392 | 214 |

Source: PASS W14-W16

**Figure A1** Results using Propensity Score Matching.

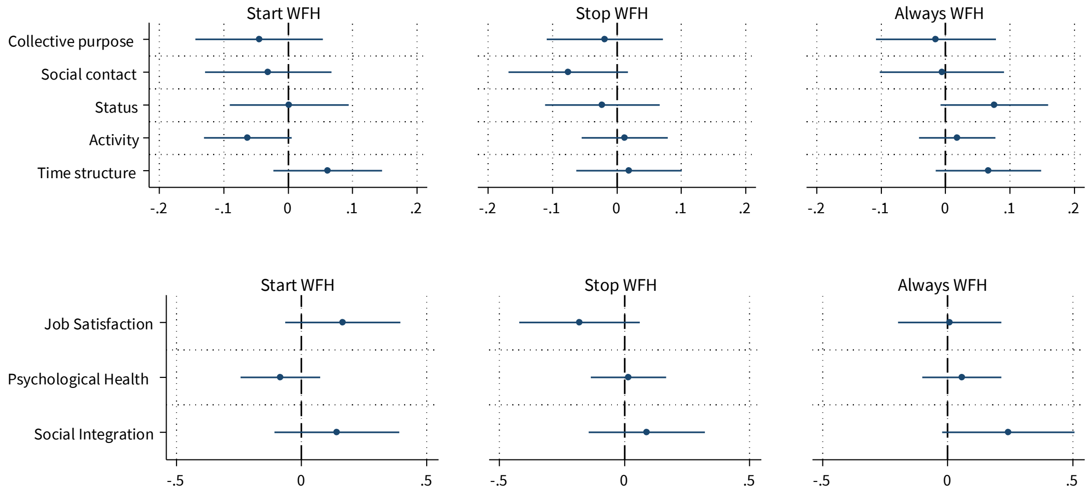


Note: Weighted OLS regression coefficients with 95% confidence intervals.
Weights caluclated using Propensity Score Matching with radius 0.01; matching calculated for each group separately vs never wfh.
Cluster-robust standard errors (two-sided test) * p < .05, ** p < .01, *** p < .001
Respondents: 2994 never wfh, 273 start wfh, 256 stop wfh, 324 always wfh
Matching variables: age, education years, income, partnership and household comp., occupation
urbanity level, industry, tenure, job change, short-time-allowance, interview mode, covid wave
Data: PASS waves 14-16 (2020-2022)

**Figure A2** Recalculation of Figure 1 for males and females

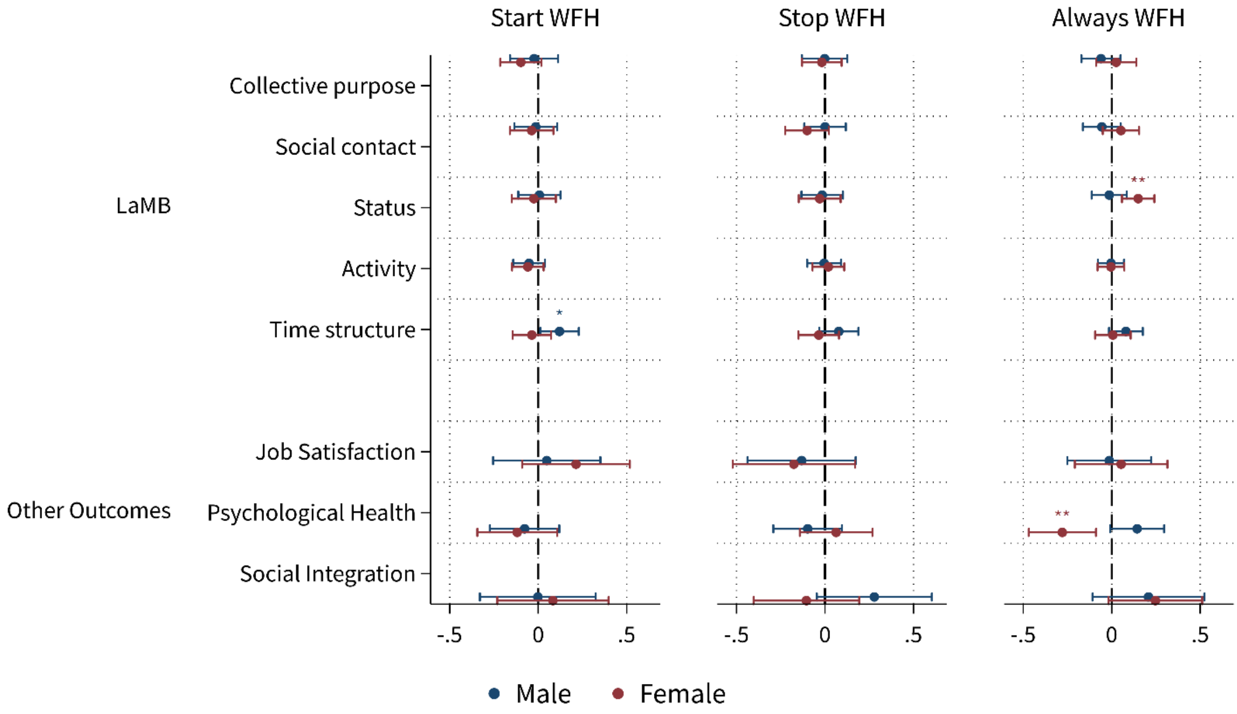

**Figure A3** Recalculation of Figure 2 for males and females

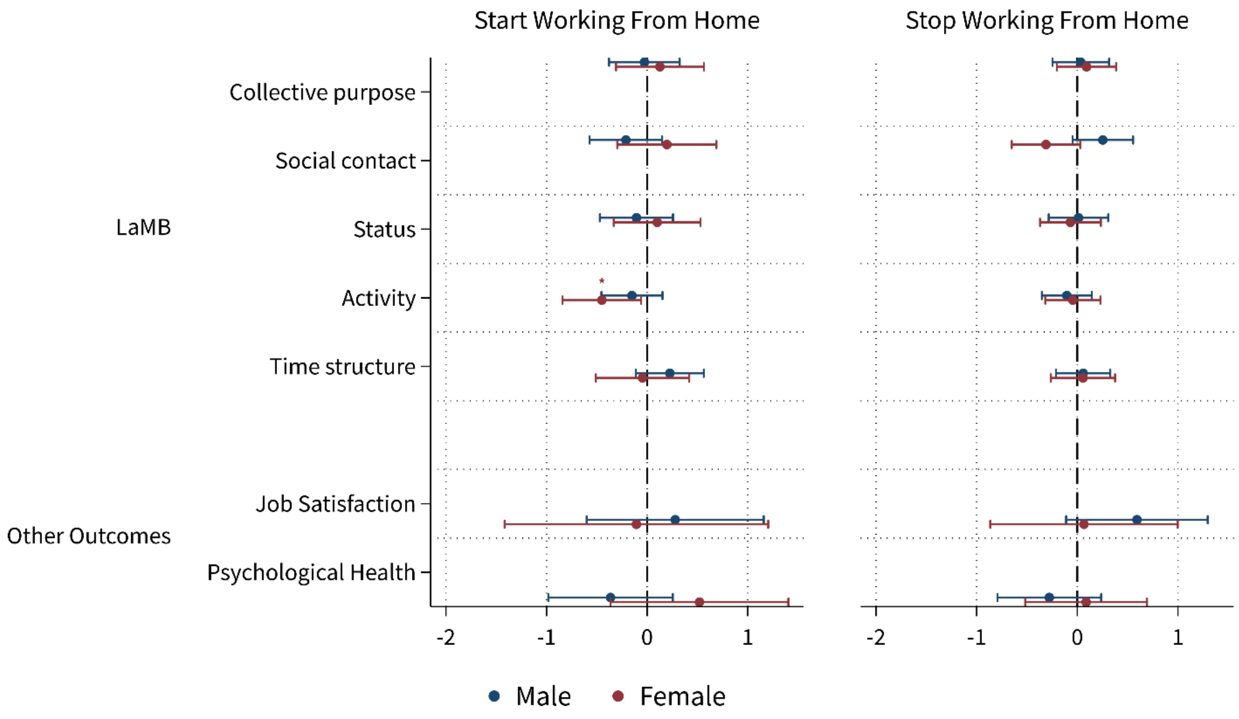


**Figure A4** Recalculation of Figure 1 for parents and non-parents

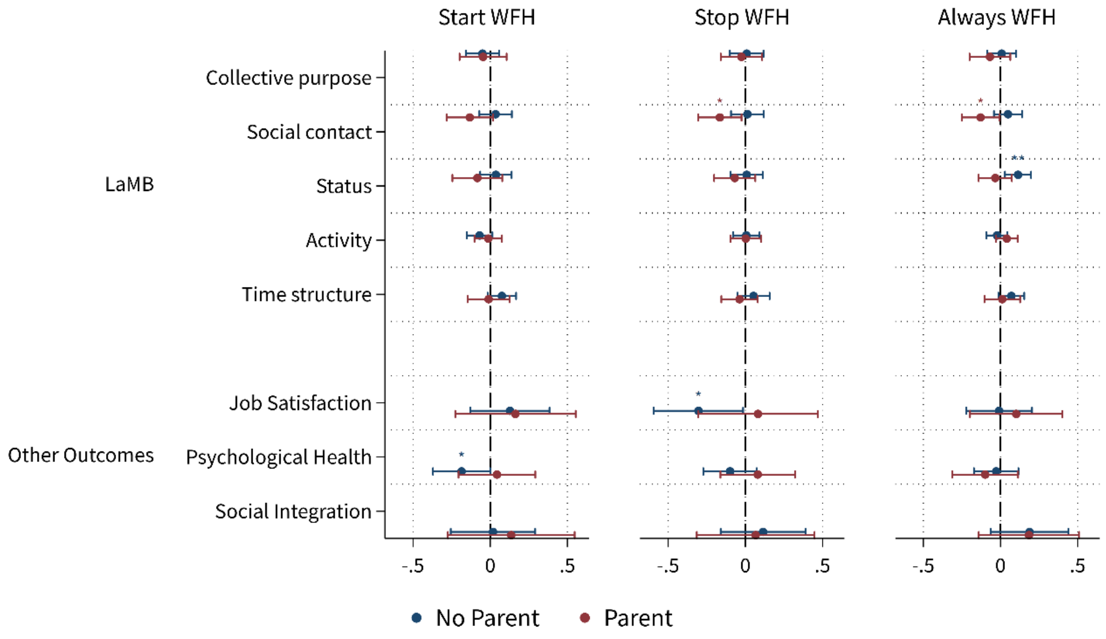


**Figure A5** Recalculation of Figure 2 for parents and non-parents

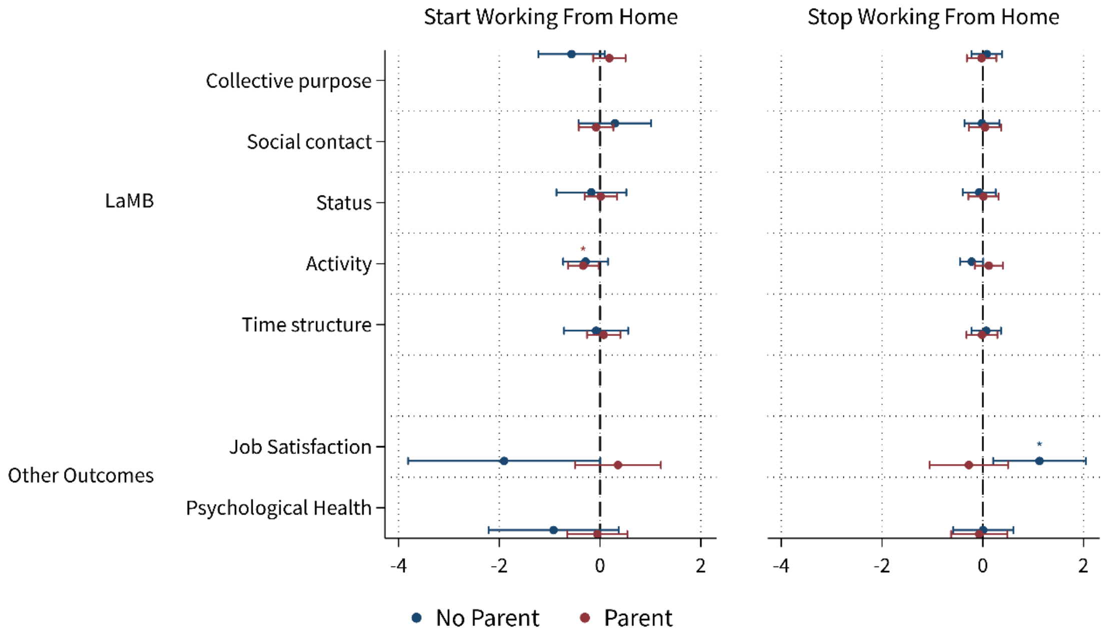


**Figure A6** Recalculation of Figure 1 for managerial and nonmanagerial positions

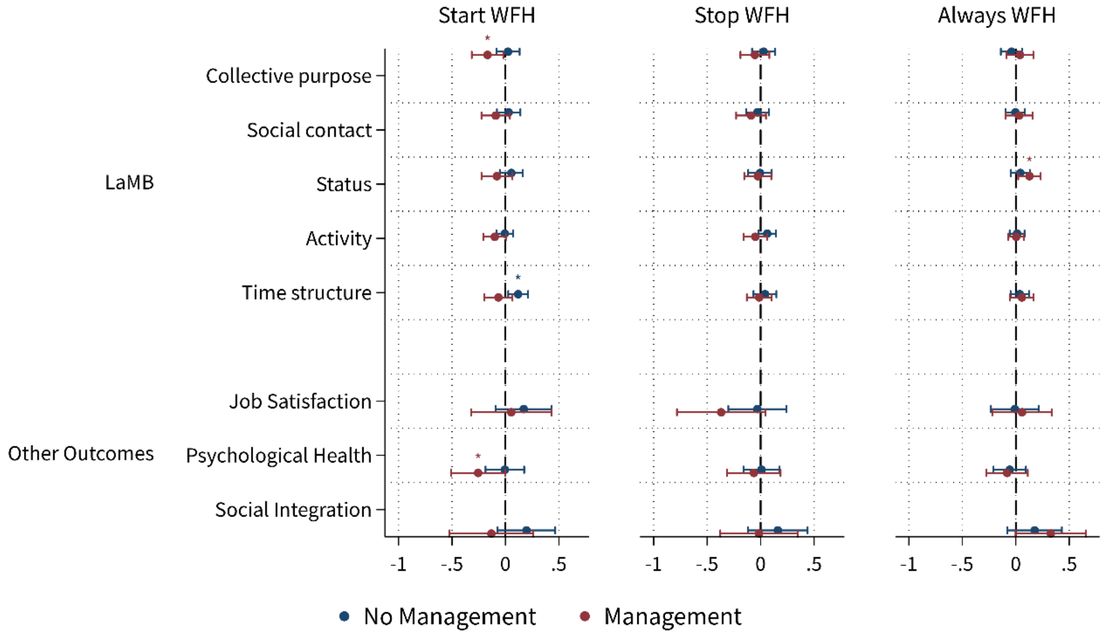


**Figure A7** Recalculation of Figure 2 for managerial and nonmanagerial positions

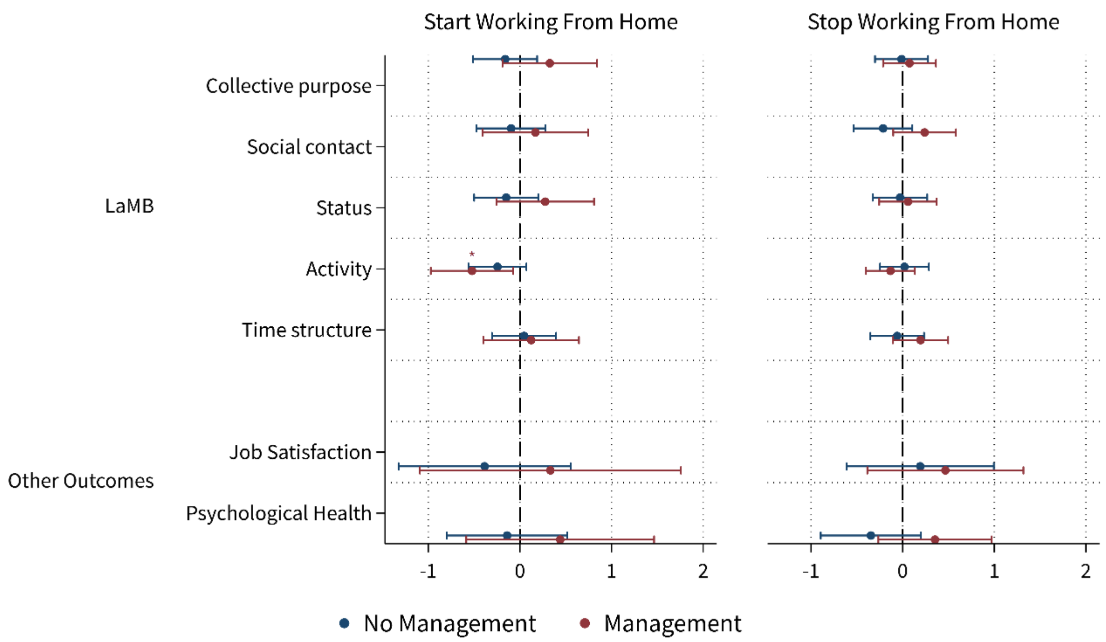

Start Working From Home
Stop Working From Home
LaMB
Collective purpose
Social contact
Status
Activity
Time structure
Other Outcomes
Job Satisfaction
Psychological Health
*
-1
0
1
2
-1
0
1
2
No Management
Management
