# Supplementary material for: Is working from home changing the meaning of work?
Source: PLoS One. 2026 Jan 27;21(1):e0340452. doi: 10.1371/journal.pone.0340452 (PMC12843579; doi:10.1371/journal.pone.0340452)
Supplement: Attachment — Submitted filename: Report PONE-D-25-30593 MB.pdf [file pone.0340452.s002.pdf]

Review Report of the paper entitled '*Is working from home changing the meaning of work?'*

This manuscript tackles an important and timely topic — the implications of working from home (WFH) on the latent benefits of employment and overall well-being, especially since the COVID-19 period. Using panel data from the German PASS survey, the authors explore whether WFH alters key psychological and structural aspects of work, such as social integration and mental health. The study's design is transparent, and the authors apply appropriate econometric tools given their stated objectives.

However, the study remains fundamentally correlational, which raises concerns about causal interpretation. While the fixed effects models partially address time-invariant confounders, the potential for self-selection into WFH and time-varying confounding is not fully accounted for. As a result, the null findings — which are interesting in themselves — could mask underlying heterogeneity or be the result of residual bias. Here below I have included some major and minor comments, which could help to improve the paper.

Major Comments:

1. *Causal Identification Strategy*. The manuscript primarily uses OLS and fixed-effects regressions to explore associations between WFH and the outcomes of interest. These are valid statistical techniques, but they do not rule out self-selection. For example, employees who choose or are chosen to work from home may differ systematically in motivation, job type, or psychological resilience. The authors should consider applying a causal inference method to strengthen the validity of their results. Potential options include: Propensity Score Matching (PSM) under the Conditional Independence Assumption (CIA) to balance observable characteristics between WFH and non-WFH groups or inverse Probability Weighting (IPW)/Doubly Robust Estimators, which would allow the authors to make better use of the panel data while adjusting for selection.
2. *Instrumental Variable based approach*, if a valid instrument such as regional lockdown intensity or broadband access is available.
3. *Unobserved Heterogeneity and Sorting*. The authors acknowledge that those who "always WFH" may be in higher-status positions, but this is not adequately addressed analytically. This group might differ in unobservable ways (e.g., job autonomy, digital skills), leading to biased estimates even in the fixed effects setup. The authors should include a more thorough discussion of the potential for time-varying unobserved confounding, and consider sensitivity checks using matching methods or subgroup stratification.
4. *Measurement of WFH Intensity*. The WFH measure is binary and does not capture the frequency or duration of remote work, which may be highly relevant for outcomes such as social contact and mental health (e.g. econometric strategy such as the generalized propensity score may help dealing with a 'treatment' which is indeed continuous in nature). The authors should clarify the limitations of the WFH variable and suggest directions for future data collection (e.g., daily or weekly frequency?).

Minor Comments

There is a typo in the main text: "comparedto" should be corrected to "compared to".
Page 3 contains a repetition: "almost almost one in four".
The introduction opens with rhetorical questions. Consider revising for a more formal tone.
The sentence “...and they deny opportunities for personal interactions...” (p. 3–4) should be revised for clarity.

Conclusion

This is a well-structured and clealy written paper on a subject of broad interest. The null results are potentially valuable, especially given the shift toward hybrid and remote work. However, the study could be significantly improved by addressing the self-selection issue and moving closer to a causal framework. With these revisions, the paper would offer a more robust contribution to the literature on remote work and worker well-being. I suggest the authors to revise and resubmit the manuscript.
