# Supplementary material for: Is working from home changing the meaning of work?
Source: PLoS One. 2026 Jan 27;21(1):e0340452. doi: 10.1371/journal.pone.0340452 (PMC12843579; doi:10.1371/journal.pone.0340452)
Supplement: Attachment — Submitted filename: response_reviewers_v4.docx [file pone.0340452.s004.docx]

**Response Letter for Manuscript "Is working from home changing the meaning of work? "**

Dear Prof. Piwowar-Sulej,

We thank you and the referees for the opportunity to revise our manuscript for further consideration for publication in PLOS One. We are grateful for the comments that helped us in improving the paper.

Below, we include a reply to all points and comments raised by the Reviewers. We display the comment as well as our response to the respective comment.

**Reviewer #1**

1. The manuscript introduces Jahoda's latent deprivation model but does not delve deeply into the psychological theories underlying it. Consider extending the theoretical framework and connecting it with contemporary perspectives in occupational or organizational psychology.

***Response:***

We thank the reviewer for this constructive comment. In the revised version, we have expanded our discussion of Jahoda's latent deprivation model by explicitly situating it within broader psychological theories. Specifically, we now connect Jahoda's framework with self-determination theory [1], conservation of resources theory [2], and the Job Demands–Resources model [3]. This extension strengthens the theoretical foundation of our study and underscores the ongoing relevance of Jahoda's model for understanding well-being in evolving work environments. We have added this point to the theoretical background in our paper.

2. The binary classification of WFH (always, never) is too simplistic. It misses the nuance in frequency (1 day/week vs. 5 days/week), voluntariness, and hybrid models. Collecting or leveraging more detailed data on WFH intensity would enhance explanatory power.

***Response:***

While we agree that this would be helpful information to have, our data set unfortunately does not provide a more fine-grained WFH intensity measure. Using WFH as a binary indicator, however, is established in the literature [4].

3. Most results are null, but there's insufficient discussion about statistical power. Are the sample sizes in subgroups (parents, managers) large enough to detect small-to-moderate effects? Include formal power calculations or at least a sensitivity analysis.

***Response:***

We thank the referee for this comment. We now show the number of observations in the newly added Appendix Table A2 and, against this backdrop, discuss statistical power issues at the end of the results section. We conclude that our sample size is large enough to exclude significant effects of WFH on the outcomes of interest.

4. Although the paper acknowledges potential self-selection into WFH, it does not explicitly model it. Consider using techniques such as instrumental variables, matching, or Heckman correction if plausible instruments or selection models are available.

***Response:***

We thank the referee for this comment. This also relates closely to the second point made by Reviewer Two, who suggests using an instrumental variable approach to exploit exogenous variation in working from home. Unfortunately, as we also point out in the response to reviewer two, we are not aware of any relevant instruments (affecting WFH propensity) and exogenous (i.e., not correlated with the outcome of interest except through WFH), as all potential instruments are also likely to affect well-being.

However, we added a propensity score matching analysis to our results in Figure A1, as this was the first point raised by Reviewer Two. In this analysis, we match on a large number of characteristics, including occupation and industry, and still find no effects of WFH on our outcomes of interest. We are thus confident that our main results are robust.

5. The annual survey waves don't capture short-term fluctuations in WFH experiences or outcomes. This temporal mismatch may attenuate observed effects. The authors should discuss this limitation more rigorously and suggest finer-grained data collection for future studies.

***Response:***

We thank the referee for this comment. Indeed, our data does not capture WFH intensity, nor whether the WFH we observe is only short-term or lasting. We agree that a more detailed look at WFH provides a promising avenue for future research and added this limitation and recommendation to our concluding section.

6. The significant finding for higher social integration among those who always WFH is speculated to be due to unobserved heterogeneity. Instead of speculation, test for such heterogeneity more systematically (by using interaction terms or stratified models).

***Response:***

We thank the referee for this comment. Those who are constantly working from home might be systematically different from those who always work in the office. Unfortunately, these differences are likely unobserved. But with the newly added results using propensity score matching (Appendix Figure A1), we show that the effect on social integration is no longer statistically significant, which we take as further evidence that there was selection that was not accounted for in the OLS regression.

7. The effects of WFH likely vary by sector (e.g., tech vs. healthcare) or job function (creative vs. administrative). The study could benefit from stratified analysis or examination of interaction effects by occupation or industry.

***Response:***

We thank the referee for this comment. Unfortunately, we lack the statistical power due to a small number of observations for specific occupations. Nevertheless, we investigate heterogeneities by managerial status in Appendix Figures A6 and A7 and show no significant differences between those with and without managerial duties. As the difference between these groups is expected to be relatively large (managing employees during WFH might be stressful), we take this, in combination with the other heterogeneities that do not show much variation, as evidence supporting our baseline results that WFH does not significantly affect well-being measures.

8. While job satisfaction is included, other aspects of job quality (autonomy, perceived fairness, career development) are not directly addressed. Consider adding these variables or discussing why they were excluded.

***Response:***

Our study focuses on the influence of WFH on the latent functions of employment, as Marie Jahoda explicitly connects these to people's well-being. To operationalize well-being, we choose mental health, social integration, and job satisfaction. Mental health features prominently in the latent deprivation model, social integration is thought to be important in relation to WFH, and job satisfaction is an umbrella measure for work-related well-being.

However, we are thankful for the reviewer's suggestion, as the PASS data provides additional concepts of well-being that also include the measures of job quality. PASS provides measures for perceived autonomy, fairness, and career prospects [5]. We recalculated the fixed effects models from Figure 02, showing these additional outcomes. Furthermore, we also included social isolation (respondents with no contacts outside their household) and general life satisfaction to broaden the focus.

From these analyses, the additional outcomes also do not change depending on whether WFH is started or stopped. To us, these null effects suggest that further studies of mediation paths using these variables are unlikely to be promising. To keep our paper's focus on the latent and manifest functions of employment, we therefore stick to our three well-being measures.

9. The manuscript claims results are likely generalizable, but cultural, regulatory, and economic differences may moderate WFH's effects. Include more comparative references or theoretical discussion on cross-national variability.

***Response:***

In our limitations section, we now make it more transparent that our results are generalizable to German office workers during the years 2020-2022. By explicitly embedding our results in the international literature on the effects of WFH on well-being, we support our claim of generalizability, given the constraints.

10. Many subgroup analyses are relegated to the appendix without interpretation in the main body. Consider integrating key findings more directly into the results and discussion sections to enhance reader engagement.

***Response:***

We thank the referee for this comment. Indeed, the heterogeneity analysis was a bit sidelined in our original submission. We have now created a new section, "Heterogeneity Analysis" as a subsection in "Results" in which we describe our heterogeneity analysis in more detail and also motivate each analysis separately.

11. The paper does not explore mediators (e.g., perceived autonomy, work-life balance) or moderators (gender, personality traits). Including these could shed light on the mechanisms or boundary conditions of WFH effects.

***Response:***

We thank the reviewer for this suggestion. As mentioned in our answer to point 8, our data allowed us to include the mentioned variables as additional outcomes of WFH. However, our analyses suggested no significant effects, which is why we refrained from more detailed causal mediation analysis and inclusion of these additional variables in the manuscript.

12. Some sections (the introduction) jump between themes, such as environmental impacts and employee well-being, without clear transitions. Streamlining the narrative around core research questions would help focus the reader.

***Response:***

We thank the reviewer for this constructive comment. In response, we have substantially revised the introduction and theoretical framing. Specifically, we shortened and condensed sections on environmental aspects, improved transitions between themes, and reorganized the structure to follow a more straightforward progression from individual experiences with WFH, to organizational and social perspectives, and finally to the theoretical framework. Finally, we concluded the introduction with an explicit formulation of our research focus. We believe that these revisions have improved the clarity, coherence, and focus of the manuscript.

**Reviewer #2**

This manuscript tackles an important and timely topic — the implications of working from home (WFH) on the latent benefits of employment and overall well-being, especially since the COVID 19 period. Using panel data from the German PASS survey, the authors explore whether WFH alters key psychological and structural aspects of work, such as social integration and mental health. The study's design is transparent, and the authors apply appropriate econometric tools given their stated objectives.

However, the study remains fundamentally correlational, which raises concerns about causal interpretation. While the fixed effects models partially address time-invariant confounders, the potential for self-selection into WFH and time-varying confounding is not fully accounted for. As a result, the null findings — which are interesting in themselves — could mask underlying heterogeneity or be the result of residual bias. Here below I have included some major and minor comments, which could help to improve the paper.

**Major Comments**:

1. *Causal Identification Strategy.* The manuscript primarily uses OLS and fixed-effects regressions to explore associations between WFH and the outcomes of interest. These are valid statistical techniques, but they do not rule out self-selection. For example, employees who choose or are chosen to work from home may differ systematically in motivation, job type, or psychological resilience. The authors should consider applying a causal inference method to strengthen the validity of their results. Potential options include: Propensity Score Matching (PSM) under the Conditional Independence Assumption (CIA) to balance observable characteristics between WFH and non-WFH groups or inverse Probability Weighting (IPW)/Doubly Robust Estimators, which would allow the authors to make better use of the panel data while adjusting for selection.

***Response:***

We thank the referee for this comment. First, we want to point out that fixed effects accounts for self-selection that is time-constant. Thus, if motivation, resilience, or job type are, at least mostly, time-constant, this type of selection is dealt with in the FE regressions. Nevertheless, we agree with the referee that there is at least some time-varying component to the described variables that could bias our estimation results. To this end, we repeated our estimations using propensity score matching, matching on age, education, household composition, occupation, industry, tenure, receipt of a short-time allowance, job changes, interview mode, and the COVID-19 wave during the time of the interview. Appendix Figure A1 shows the results. Again, we find no effects of WFH on our outcomes of interest. As we can produce these results using three different estimation approaches, we are confident that the results are robust.

2. *Instrumental Variable based approach*, if a valid instrument such as regional lockdown intensity or broadband access is available.

***Response:***

We thank the referee for this comment. Unfortunately, we are not aware of any valid instrument that we could use for our analysis. The instrument has to be i) relevant, i.e., correlate with WFH significantly, and ii) exogenous, i.e., affect the outcomes of interest only through WFH and not directly or through a confounder. Unfortunately, the instruments that we could think of, such as broadband access and regional lockdowns, are likely relevant but also not exogenous. Broadband access is also likely to affect the outcomes of interest directly, for example, by enhancing well-being through broader access to entertainment services. Lockdowns are also expected to affect well-being directly, as individuals may be afraid of infection if local incidence rates are high.

The literature sometimes uses instruments such as the employer's digital infrastructure before COVID to instrument WFH, but we do not have information on these characteristics in our data. Thus, unfortunately, we cannot think of an instrument that is relevant, exogenous, and available in our data.

3. *Unobserved Heterogeneity and Sorting.* The authors acknowledge that those who "always WFH" may be in higher-status positions, but this is not adequately addressed analytically. This group might differ in unobservable ways (e.g., job autonomy, digital skills), leading to biased estimates even in the fixed effects setup. The authors should include a more thorough discussion of the potential for time-varying unobserved confounding, and consider sensitivity checks using matching methods or subgroup stratification.

***Response:***

In our analysis, we first present OLS results to establish the different WFH groups, and also include individuals who consistently worked from home during the observation window. While we argue that starting and always WFH suffered less from sorting during the COVID-19 pandemic, sorting can nonetheless be an issue. To ascertain the extent of sorting, we added a propensity score matching analysis to our results in Figure A1. In this analysis, we match on many characteristics, including occupation and industry, and still find no effects of WFH on our outcomes of interest. We are thus confident that our main results are robust.

Our fixed effects longitudinal analysis, which relies only on within-individual changes, should provide more robust results. Remaining sorting and heterogeneity thus need to be time-varying to bias our results. And while we control for a host of sociodemographic dynamics and job changes, we lack information on more subtle work-related changes, such as workplace policies (e.g., changes in job autonomy) or the career prospects of remote workers compared to those of onsite workers. These factors might influence both an office worker's decision on where to work and their overall well-being. We acknowledge this now explicitly in our limitations section.

4. *Measurement of WFH Intensity.* The WFH measure is binary and does not capture the frequency or duration of remote work, which may be highly relevant for outcomes such as social contact and mental health (e.g. econometric strategy such as the generalized propensity score may help dealing with a 'treatment' which is indeed continuous in nature). The authors should clarify the limitations of the WFH variable and suggest directions for future data collection (e.g., daily or weekly frequency?).

***Response:***

Unfortunately, there is no WFH intensity available in our data. We added this point as a limitation and an avenue for future research to the discussion at the end of our manuscript.

**Minor Comments**

There is a typo in the main text: "comparedto" should be corrected to "compared to". Page 3 contains a repetition: "almost almost one in four".

The introduction opens with rhetorical questions. Consider revising for a more formal tone. The sentence "...and they deny opportunities for personal interactions..." (p. 3–4) should be revised for clarity.

***Response:***

Thank you. We changed the text accordingly.

**Conclusion**

This is a well-structured and clearly written paper on a subject of broad interest. The null results are potentially valuable, especially given the shift toward hybrid and remote work. However, the study could be significantly improved by addressing the self-selection issue and moving closer to a causal framework. With these revisions, the paper would offer a more robust contribution to the literature on remote work and worker well-being. I suggest the authors to revise and resubmit the manuscript.

References

**1**.	Ryan RM, Deci EL. Self-determination theory and the facilitation of intrinsic motivation, social development, and well-being. Am Psychol. 2000; 55:68–78. doi: 10.1037/0003-066X.55.1.68 PMID: 11392867.

**2**.	Hobfoll SE. Conservation of resources. A new attempt at conceptualizing stress. Am Psychol. 1989; 44:513–24. doi: 10.1037/0003-066X.44.3.513 PMID: 2648906.

**3**.	Bakker AB, Demerouti E. The Job Demands‐Resources model: state of the art. Journal of Managerial Psych. 2007; 22:309–28. doi: 10.1108/02683940710733115.

**4**.	Senik C, Clark AE, D'Ambrosio C, Lepinteur A, Schröder C. Teleworking and life satisfaction during COVID-19: the importance of family structure. J Popul Econ. 2024; 37. doi: 10.1007/s00148-024-00979-z.

**5**.	Müller B, Bähr S, Gundert S, Teichler N, Unger S, Wenzig C. PASS Scales and Instruments Manual. ; 2020.
