# Supplementary material for: Is working from home changing the meaning of work?
Source: PLoS One. 2026 Jan 27;21(1):e0340452. doi: 10.1371/journal.pone.0340452 (PMC12843579; doi:10.1371/journal.pone.0340452)
Supplement: Attachment — Submitted filename: response_editor.docx [file pone.0340452.s005.docx]

**Response Letter for Manuscript "Is working from home changing the meaning of work?" (Manuscript PONE-D-25-30593R1)**

Dear Prof. Piwowar-Sulej,

We thank you for the opportunity to revise our manuscript for further consideration for publication in PLOS One. We are grateful for the comments that helped us in improving the paper.

Below, we include a reply to all points and comments raised. We display the comment as well as our response to the respective comment.

After a careful evaluation of the submitted manuscript, I must note that the document in its current form does not meet the standards of a scientific article. Instead, it resembles a research report. Substantial revisions are necessary before the manuscript can be considered for publication. Below I outline the key issues.

1. **Lack of academic article structure**

The Introduction fails to identify a clear **research gap**. It remains unclear what has already been studied in the field and what specific gap the present study aims to address. In journals such as PLOS ONE, authors are required to define the context, the problem, and the research gap clearly so that the contribution of the article is explicit.

***Response:*** *Indeed, the research gap was not clear in the last round of revisions. To this end, we completely rewrote the introduction and now clearly state that we are the first ones to apply Jahoda’s concept of latent functions of work to the transition from working in office to working from home and vice versa using state-of-the art empirical estimation strategies to come as close as possible to identifying a causal effect of working from home on the respective factors.*

2. **Missing theoretical framework**

The manuscript does not follow the conventional structure (Introduction, **Literature review**, Methods, Results, Discussion). Most importantly, the manuscript does not contain a theoretical section that would allow the development of hypotheses or research questions. **As a result, the empirical part appears disconnected from any conceptual background.**
A valid empirical study must be grounded in theory; otherwise, the rationale behind the research design and hypotheses remains unclear.

***Response:*** *We added a new Section (Section 2) that provides a theoretical discussion of how Jahoda’s (and comparable concepts) can be used as a theoretical basis to derive specific research questions, which we do in the revised version of the manuscript.*

3. **Insufficient discussion of findings**

The **Discussion section should compare the findings with previous research**. In the current version, the authors do not relate their results to similar studies conducted by other scholars.
Moreover, the manuscript fails to articulate its **contribution to theory and practice** in the field of management. This must be presented explicitly and supported with argumentation.

***Response:*** *In Section 5 (Discussion), we embed our findings within the existing literature and tie them back to our specific research questions Furthermore, in the ultimate paragraph of Section 5, we now provide a clear implication for practice: whether work is performed in-office or from home has no effect on employee’s well-being.*
